# Prevalence of Intestinal Parasitic Infections among Children in Europe over the Last Five Years

**DOI:** 10.3390/tropicalmed6030160

**Published:** 2021-09-02

**Authors:** Maria Kantzanou, Maria A. Karalexi, Georgia Vrioni, Athanasios Tsakris

**Affiliations:** 1 Department of Hygiene, Epidemiology & Medical Statistics Medical School, National and Kapodistrian University of Athens, 75 Mikras Asias, 11527 Athens, Greece; maria.kantzanou@gmail.com; 2 Laboratory of Microbiology, Medical School, National and Kapodistrian University of Athens, 75 Mikras Asias Street, 11527 Athens, Greece; gvrioni@med.uoa.gr (G.V.); atsakris@med.uoa.gr (A.T.)

**Keywords:** intestinal parasitic infections, *Blastocystis*, children, Europe, prevalence, meta-analysis

## Abstract

While the prevalence of intestinal parasitic infections (IPI) has been most commonly studied in African and Asian populations, less is known about the prevalence rates of IPI in European children, as well as the potential risk factors that favor the spread of parasites. We aimed to review published evidence on the prevalence rates of IPI in children residing in Europe, and to quantitatively synthesize the results of published studies. We searched Medline from 1 January 2015 to 1 April 2021 to address the most recently published prevalence patterns of IPI in European children. Random-effects meta-analyses were performed by type of IPI infection, age group and sex, depending on data availability. Of the 967 potentially relevant articles, eight eligible cross-sectional studies were included in this analysis, yielding a sample of 3376 children (0–19 years). The overall prevalence rate was 5.9% for any IPI in children residing in European countries. *Blastocystis hominis* was the most commonly detected parasite yielding a prevalence rate of 10.7%. Other parasites included *Entamoeba coli, Endolimax nana,* and *Blastocystis hominis*. Studies focusing on specific types of parasites showed prevalence rates ranging from 1.3% for *Cryptosporidium* to 68.3% for *Dientamoeba fragilis*. Despite the scarce literature, the present review showed relatively low prevalence rates of IPI in Europe. Future studies accounting for proper diagnostic methods used for the detection of parasites and including information on potential sociodemographic factors, such as travelling history and history of immigration, are needed to guide clinicians about which children to test, as well as when and how to test children for IPI.

## 1. Introduction

Intestinal parasitic infections (IPI) in children are highly prevalent in regions with limited or no access to safe drinking water, poor sanitation and substandard housing conditions [1]. Around 24% of the global population are infected with soil-transmitted helminth infections, of which more than 267 million preschool-age children, and over 568 million school-age children live in epidemic areas [2]. In African countries, such as Ethiopia, the prevalence rate of IPI in children is estimated to be 48% (95% confidence intervals [CI]: 42–53%) [3]. The majority of infections are caused by *Ascaris lumbricoides*, hookworm, and *Trichuris trichiura* [4]. *Cryptosporidium species, Entamoeba histolytica and Giardia duodenalis* are the most common protozoan infections in children aged under 5 years in sub-Saharan Africa [4].

There are significant concerns about the potential short-term and long-term complications of IPI in children [5]. Parasites may cause malabsorption and chronic blood loss, with long-term effects on the physical and cognitive development of children [6,7]. Especially in disadvantaged populations, malnutrition renders children more vulnerable to IPI, which in turn may result in protein-energy malnutrition, iron-deficiency anemia and subsequent deficits in both mental and physical growth [6].

While the prevalence and time trends of intestinal parasitic infections have been more commonly studied in African and Asian populations, far less is known about the prevalence rates of these infections in Europe [1,8]. Few studies have examined the incidence patterns of IPI in European populations, as well as the potential risk factors that favor the spread of parasites in children. There is mounting evidence that socio-economic factors adversely affect health status, and may favor environmental fecal contamination and interpersonal transmission of direct-cycle parasites even in a developed country [8,9], the majority of previous studies have reported high prevalence of IPI in immigrants (20.8%) as well as in vulnerable populations, such as patients hospitalized for mental health illnesses (55.5%) [10,11,12]. By contrast, recent studies have shown that the interpersonal transmission of the direct cycle of other parasites, such as *Enterobius vermicularis* in European children is not strongly associated with socio-economic factors and environmental fecal contamination. Overall, few reviews have comprehensively synthesized the existing evidence in Europe in order to guide future avenues by assisting the public health authorities to implement better control strategies.

We aimed to review published evidence on the prevalence rates of IPI in children residing in Europe, and to quantitatively synthesize the results of published studies. Whenever possible, separate analyses were performed by type of IPI infection, age group and sex aiming to shed light into the global prevalence of IPI among European children.

## 2. Results

### 2.1. Characteristics of the Studies

**Figure 1:**
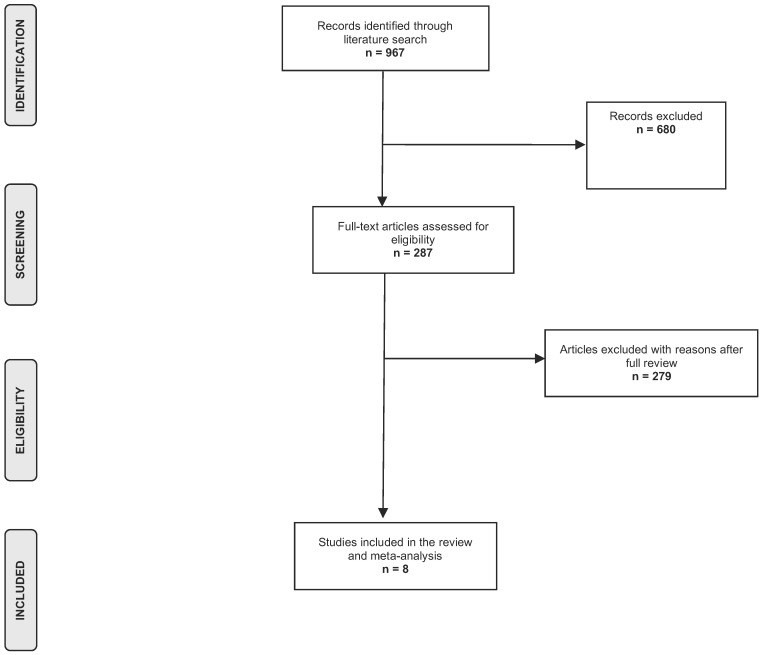
Flow chart of the literature search process.

Figure 1 shows the results of the literature search and selection process. The initial database search identified 967 potentially relevant articles. No articles were identified through snowball sampling. Following the titles and abstract screening, 680 publications were excluded. Of the remaining publications, 279 studies were excluded for specific reasons, namely studies focused on Asian, American or African populations, reviews, animal or experimental studies. Thus, eight eligible studies were finally included in this analysis [13,14,15,16,17,18,19,20].

The descriptive characteristics of the included studies are presented in Table 1. All studies were cross-sectional yielding a total size of 3376 children aged 0–19 years, while one study examined the prevalence of IPI in children 0–7 years old [14]. The average age of participants ranged between 2.8 and 8.4 years. In all studies, parasites were detected in stool samples, whereas in one study both blood and stool tests were performed [19].

### 2.2. Prevalence of Intestinal Parasitic Infections

Six studies examined the prevalence of specific types of parasites in children, namely Enterobius vermicularis [14], Strongyloides stercoralis [19], Blastocystis [13,20], Dientamoeba fragilis [15], Giardia duodenalis [16] and Cryptosporidium [16].

The remaining two studies examined any type of intestinal parasite detected in children’s stool samples [17,18]. Specifically, a recent Swiss study showed a prevalence rate of 4.2% for *Blastocystis hominis* detected in children’s stool samples, whereas co-infection with one (0.7%) or several (0.3%) other parasites was also identified, of which the most common was *Endolimax nana* (0.5%) [18]. The second study in Slovakia identified *Entamoeba coli, Endolimax nana,* and *Blastocystishominis* in stool samples performed in regions where inhabitants mostly live in settlements with low levels of hygiene. Of note was the higher prevalence of infections with *Endolimax nana* in children 8–18 years compared to older and younger age groups (*p* = 0.0498) [17].

**Figure 2:**
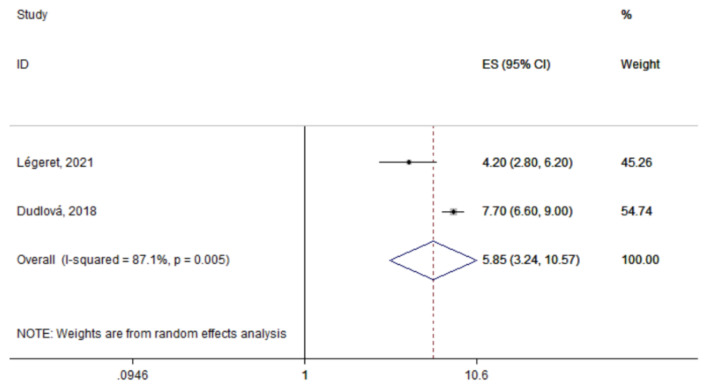
Forest plot of the summary prevalence of intestinal parasitic infections in children residing in Europe (2015–2021). Prevalence ratios of individual studies are indicated by the data markers; shaded boxes around data markers reflect the statistical weight of the study; 95% confidence intervals (CI) are indicated by the error bars; summary-effect estimates with their 95% CI are depicted as a diamond.

**Figure 3:**
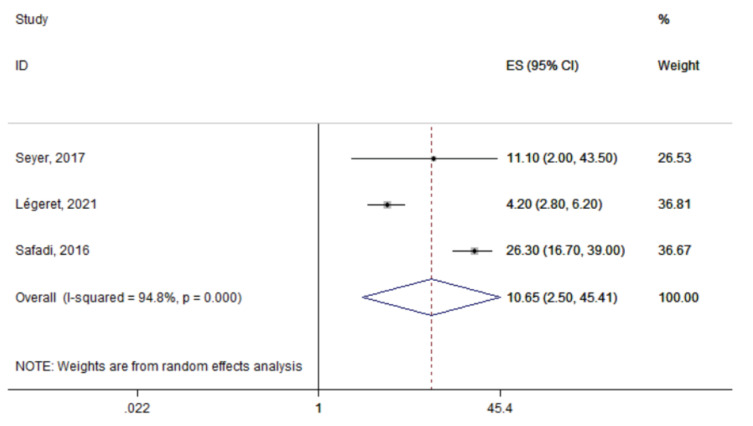
Forest plot of the summary prevalence of *Blastocystis hominis* infections in children residing in Europe (2015–2021). Prevalence ratios of individual studies are indicated by the data markers; shaded boxes around data markers reflect the statistical weight of the study; 95% confidence intervals (CI) are indicated by the error bars; summary-effect estimates with their 95% CI are depicted as a diamond.

The summary prevalence of IPI was estimated at 5.9% (95% CI: 3.2–10.6%) based on the two studies that assessed any type of parasites in children’s stool samples (Figure 2). However, heterogeneity was significant (I^2^: 87.1%; *p* = 0.005). The most commonly detected parasite across the identified studies was *Blastocystis species* with a summary prevalence of 10.7% (95% CI: 2.5–45.4%; n = 3 studies; Figure 3). Again, significant between-study heterogeneity was found (I^2^: 94.8%; *p* < 0.0001)

## 3. Discussion

### 3.1. Main Findings

The present review of approximately 1000 studies published during the last five years showed a prevalence rate of 5.9% for any IPI in children residing in European countries. Despite the abundance of evidence on IPI in Asian and African populations, recent literature about the prevalence of such infections in Europe is scarce. Our literature search yielded eight eligible studies on our research question. Among specific types of parasites, *Blastocystis hominis* was the most commonly detected type, yielding a prevalence rate of 10.7%. Other parasites included *Entamoeba coli, Endolimax nana,* and *Blastocystishominis*. Studies focusing on specific types of parasites, such as *Enterobius vermicularis, Strongyloides stercoralis, Blastocystis, Dientamoeba fragilis* and *Giardia duodenalis* showed prevalence rates ranging from 1.3% for *Cryptosporidium* to 68.3% for *Dientamoeba fragilis*.

### 3.2. Interpretation of Findings

The overall pooled prevalence of IPI in the present systematic review and meta-analysis is much lower than that of Asian and African countries, such as Ethiopia (48%) [3], Nigeria (54.8%) [21], Rwanda (50.5%) [22], Iran (38%) [23] and Turkey (31.7–37.2%) [24]. Worldwide, areas with high rates of IPI include India, Africa, and Central and South America. Such discrepancies in prevalence rates have been attributed to socioeconomic factors, poor hygiene and sanitary facilities, weather, climate and environmental factors. Several types of parasites most commonly affect children living under specific conditions [25]. For example, Ascaris lumbricoides infections seem to be more common in children living in households with lower incomes (prevalence ratio: 6.68, 95% CI: 1.01–44.34), while Giardia lamblia infections predominate in households with an unprotected water source (prevalence ratio: 1.95, 95% CI: 0.96–3.99) [26]. Moreover, specific lifestyle and nutritional habits, such as consuming uncooked meat, which is very common in Ethiopian communities, might increase the risk of exposure to human helminths [27]. In addition, studies have reported that travelers to low and middle-income countries (mainly areas in South America, Africa and South Asia) experience between a 9- and 151-times higher risk of developing IPI [28,29]. A recent Swiss study identified in the present review showed that 80% of patients with a negative stool sample did not have a history of travelling abroad [18].

Beyond socioeconomic status, demographic factors, such as age and sex, have also been associated with distinct patterns of prevalence of IPI. Indeed, the present review identified one study that showed higher prevalence of IPI caused by *Endolimax nana* in children 8–18 years compared to younger ones. Previous studies have also shown higher prevalence of IPI in school-age (52%) compared to preschool-age children (30%) [30,31,32]. A potential explanation may be due to the habits of playing with or handling of contaminated soils, eating with soiled hands, unhygienic toilet practices, the drinking and eating of contaminated water and food in school-age children compared to preschool-age children, who are usually cared for in-family. The studies identified in the present review did not show any sex-specific differences in the prevalence of IPI. Some previous reports in African and Asian children infected by intestinal parasites have provided evidence for a potential predominance of these infections in girls, which may be due to different nutritional habits than that of boys [23]. However, the literature remains inconclusive in terms of any sex-specific prevalence pattern of IPI in children [33].

In the present review, the most prevalent type of parasite detected across the identified studies were *Blastocystis* species. Consistent with the present findings, reports from other industrialized countries, such as Denmark and the Netherlands, have also shown a predominance of *Blastocystis* species infections, with prevalence rates of 5.6% [34] and 20% [35], respectively. A multicenter study in Izmir, Turkey also showed predominance of *Blastocystis hominis* (14.6%) followed by *Enterobius vermicularis* (10.1%) and *Giardia intestinalis* (7.8%) [36]. Blastocystis species, *Entamoeba coli* and *Giardia duodenalis* infections are mainly related to ingestion of food or water contaminated by feces, and are epidemic in underprivileged populations [37]. In children with diarrhea, *Giardia* is considered the most common culprit and may lead to malabsorption and nutritional deficiencies that may impair the child’s growth and development [38,39].

### 3.3. Strengths and Limitations

The results of the present review and meta-analysis should be cautiously interpreted in view of limitations inherent to the data availability and the large heterogeneity in the methodology and exposure assessment across the eligible studies. Moreover, six of the eight identified studies focused on specific types of parasites, the majority of which on *Enterobius vermicularis,* thus not allowing for a quantitative synthesis of the results in order to calculate the overall summary prevalence of IPI across European countries. Indeed, this data compilation does not reflect the real picture of parasitic infections in children in Europe. The limited number of studies published between 2015 and 2021 that examined the prevalence of IPI in Europe is another limitation, which did not allow the generalizability of the results of the present review. A future study is planned evaluating the prevalence rates of IPI during the last 15 years in order to allow comparisons with the most recently published evidence. Lastly, we should acknowledge the limitations of the methodologies used, the specific professional training and the specific stool methods that need to be used for the diagnosis of some parasites, such as hookworms and pinworms. Indeed, in the majority of studies, a single stool sample examination was used, which may have led to over- or underestimation of the true prevalence. The current guidelines the use of suggest multiple, at least three, stool samples for the accurate diagnosis of IPI [40].

Beyond these limitations, the sound methodological approach is a strength of the current study. We also attempted to separately present the results of the identified studies by type of parasite, age and sex.

## 4. Methods

### 4.1. Search Strategy and Study Selection

A literature search of Medline database was conducted from 1 January 2015 up to 1 April 2021 following the Preferred Reporting Items for Systematic Reviews and Meta-Analyses (PRISMA) guidelines (Supplementary Table S1) [41].

Two reviewers performed the literature search independently and blindly to each other using an algorithm combining relative key terms, such as “intestinal parasitosis”, “parasitic infection”, “parasite”, “children”, “prevalence”, “risk factors” and “Europe”. Reference lists of all relevant reviews and identified eligible studies were additionally hand-searched for potentially eligible articles through a “snowball” procedure [41].

Eligible studies were articles that examined the prevalence rates of intestinal parasitic infection in children residing in Europe. The search was limited to articles published during the last five years (2016–2021) in order to address the most recently published prevalence patterns of IPI in European children. No language or other restrictions were applied. Case reports, experimental or animal studies were excluded.

Following the literature search, duplicate citations were removed and the remaining articles were independently screened by two investigators to identify studies that met the pre-determined inclusion criteria. The study selection was conducted in two stages. Firstly, the identified studies were assessed on the basis of titles and/or abstracts; those clearly not relevant to the objective of the current review, as well as those failed to meet one or more of the selection criteria, were excluded. For the remaining studies, the full-papers were retrieved for further screening. In case of disagreement in the selection of studies or snowball procedure, the final decision was reached by team consensus. In articles with overlapping populations, the most recent or most complete publication was considered eligible.

### 4.2. Data Extraction

For each eligible publication, the following study variables were extracted: publication year, location, study design and study period, sample size, age at diagnosis, and the proportion of males. In addition, information about the number of children with IPI and the number of children with no infection was extracted. The data extraction was performed by two reviewers and any disagreements were resolved by consensus.

### 4.3. Statistical Analysis

A descriptive presentation of the eligible studies was initially performed (Table 1). Thereafter, the prevalence of IPI and 95% CI were extracted or calculated from the available data using the Wilson’s method [42]. Separate meta-analyses were performed by type of IPI infection, age group and sex, depending on the availability of data. Meta-analyses were undertaken using random-effects models [43]; between-study heterogeneity was assessed using the Cochran Q and I^2^ statistics. The Z-test was applied for the overall effect and statistical significance was set at *p* < 0.10.

Analyses were performed using the Stata software.

## 5. Conclusions

Despite the scarce literature regarding helminth and parasitic infections amongst European children, the present review showed relatively low prevalence rates of IPI. Heterogeneity issues and diverse study settings should be taken into account when interpreting the results of the present study. Future studies accounting for proper diagnostic methods used for the detection of parasites and including information on potential sociodemographic factors, such as travelling history and history of immigration, are needed to allow firm conclusions to be drawn in regard to the true prevalence of IPI in Europe and to guide clinicians about which children to test, as well as when and how to test them.

## Figures and Tables

**Table 1:** Descriptive characteristics and main findings of eligible studies.

Author, Year	Study Design	Study Period	Region	Age at Diagnosis (Years)	Sample Size	Percentage of Males	Exposure Ascertainment	Prevalence Rate of IPI	Type of Parasites Detected
Légeret, 2021	Cross-sectional	2008–2018	Switzerland	0–18 (median: 7.9)	572	44.0%	Children under the care of the Children’s hospital Aarau whose stool was tested in the last 10 years for parasites and helminths	4.2%	-*Blastocystis hominis* (4.2%); Co-infection with one (0.7%) or several (0.3%) other parasites, of which the most common *Endolimax nana* (0.5%) −80% of negative stool samples associated with no history of travelling abroad
Patsantara, 2016	Cross-sectional	2007–2009	Greece	1–18 (median: 8.39)	215	NR	Examinations conducted using the Graham test for the microscopic detection of Enterobius vermicularis eggs in the perianal area	7.7%	*Enterobius vermicularis* (7.7%)
Dudlová, 2018	Cross-sectional	NR	Slovakia	0–18	2000	42.5%	Sampling of stool performed from regions where inhabitants mostly live in settlements with a low level of hygiene (Roma settlements)	2.0%	*Entamoeba coli* (0.9%); *Endolimax nana* (0.6%); *Blastocystis hominis* (0.6%) -Higher prevalence of infection with *Endolimax nana* in children 8–18 years compared to other age groups (*p* = 0.0498)
Štrkolcová, 2017	Cross-sectional	2013–2015	Slovakia	1–17	81	49.4%	Children underwent a parasitol respreventive medical exam carried out by the pediatrician at the healthcare facility in Medzev, who performed blood and stool collection	30.9%	*Strongyloides stercoralis* (30.9%)
Seyer, 2017	Cross-sectional	NR	North Cyprus	7–19	9	NR	Stool samples collected from both asymptomatic and symptomatic volunteers	11.1%	*Blastocystis hominis* (11.1%)
Jokelainen, 2017	Cross-sectional	2009–2012	Denmark	0.9–6.6 (median, 2.8)	138	56.5%	Stool samples alongside questionnaires completed by the parents or guardians	68.3%	*Dientamoeba fragilis* (68.3%) -Age >3 years and having a history of recent traveling abroad as risk factors for testing positive for *D. fragilis*
Safadi, 2016	Cross-sectional	2012–2013	France	0–14	57	NR	Stool samples tested for *Blastocystis* sp. by quantitative PCR targeting the SSU rDNA gene	26.3%	*Blastocystis hominis* (26.3%)
Skovgaards, 2018	Cross-sectional	20052015	Denmark	0–16	304	NR	Samples analyzed by in-house real-time PCR for *Cryptosporidium* sp. and Giardia duodenalis	1.3%	*Giardia duodenalis* (0.0%); *Cryptosporidium* (1.3%)

Abbreviations: IPI, intestinal parasitic infection; NR, not reported.

## Data Availability

Not applicable.

## Supplementary Materials

The following are available online at https://www.mdpi.com/article/10.3390/tropicalmed6030160/s1, Table S1: Preferred Reporting Items for Systematic Reviews and Meta-Analyses (PRISMA) checklist.
